# A Longitudinal Study of the Relationship Between Lower Extremity Field Tests and Medial Elbow Injuries in Elementary School Baseball Players

**DOI:** 10.3390/ijerph21111518

**Published:** 2024-11-14

**Authors:** Maki Tanaka, Takuma Okutani, Shohei Maruyama, Kenji Suehiro, Tomoyuki Matsui, Machiko Hiramoto, Yoshikazu Azuma, Tetsuya Miyazaki, Megumi Gonno, Teruo Nomura, Kyosuke Oku, Yuya Watanabe, Yoshihiro Kai, Minoru Takeshima, Toru Morihara, Noriyuki Kida

**Affiliations:** 1 Department of Childhood Education, Faculty of Human Development and Education, Kyoto Tachibana University, 34 Yamada-cho, Oyake, Yamashina-ku, Kyoto 607-8175, Japan; 2 Kyoto Tanabe Central Hospital, Medical Corporation Sekitetsukai, 6-1-6 Tanabechuo, Kyotanabe-shi 610-0334, Japan; oktn1017tkm@gmail.com (T.O.); baseball.615.smile@gmail.com (S.M.); suehiro@sekitetsukai.or.jp (K.S.); mt30r7033@gmail.com (M.T.); 3 Corporate Headquarters, Medical Corporation Sekitetsukai, 6-1-6 Tanabechuo, Kyotanabe-shi 610-0334, Japan; 4 Marutamachi Rehabilitation Clinic, 12 Nishinokyo Kurumazakacho Nakagyo-ku, Kyoto 604-8405, Japan; matsui.tomoyuki.sports.reha@gmail.com (T.M.); true.to.your.heart810@gmail.com (M.H.); azuma.yoshikazu.reha@gmail.com (Y.A.); mtsports0512@gmail.com (T.M.); toru4271@koto.kpu-m.ac.jp (T.M.); 5 Department of Childhood Education, Faculty of Childhood Education, Nagoya Women’s University, 3-40 Shioji-cho, Mizuho-ku, Nagoya-shi 467-8610, Japan; gonno@nagoya-wu.ac.jp; 6 Faculty of Arts and Sciences, Kyoto Institute of Technology, Hashikami-cho, Matsugasaki, Sakyo-ku, Kyoto 606-8585, Japan; note0420@gmail.com (T.N.); kyosuke.oku@gmail.com (K.O.); kida@kit.ac.jp (N.K.); 7 Department of Sports Study, Faculty of Sport Study, Biwako Seikei Sport College, 1204 Kitahira, Otsu-shi 520-0503, Japan; watanabe-yuy@bss.ac.jp; 8 Department of Physical Therapy, Faculty of Health Sciences, Kyoto Tachibana University, 34 Yama-da-cho, Oyake, Yamashina-ku, Kyoto 607-8175, Japan; kai-y@tachibana-u.ac.jp

**Keywords:** field tests, deep squatting test, medial elbow injuries, baseball player, longitudinal study

## Abstract

This study aimed to examine prospectively the relationship between the deep squatting test and medial elbow injuries in elementary school baseball players and to determine the usefulness of the lower extremity field test in throwing injuries. The participants were 254 players who had participated in preseason medical checkups in both 2019 and 2020 and had no problems with the 2019 medical checkups. The evaluation parameters included ultrasonography examination, physical examination, and three types of deep squatting tests. The success rate of deep squatting tests was significantly lower in the injured group than in the non-injured group in the medial elbow injuries. By the difficulty level, the backward test in the deep squatting test was more likely to predict medial elbow injuries than the forward and middle tests. Furthermore, there was a significant difference in the squatting test and medial elbow injuries by grades. In conclusion, this is the first prospective study to provide evidence that the deep squatting tests, classified by difficulty level, can predict the risk of subsequent medial elbow injuries in elementary school players. The deep squatting test may be useful as a field test for lower extremity flexibility that can be performed by athletes and instructors, as it can predict the risk of subsequent medial elbow injuries.

## 1. Introduction

The early detection and prevention of sports injuries play a critical role in reducing sports injuries and enhancing exercise performance. Field tests have been used for screening to predict sports injuries [1,2,3,4,5,6,7]. The field test is a simple test to predict the risk factors for sports injuries from physical conditioning. For baseball players, shoulder and elbow injuries are major problems, and the development of tests for early detection is necessary.

Previous studies have reported that risk factors for shoulder and elbow pain and injuries are decreased range of motion (ROM) and side-to-side differences in horizontal adduction in the shoulder, lack of shoulder external rotation, preseason total shoulder rotation deficit, and muscle strength of external rotation of the shoulder [8,9,10,11,12,13]. With regard to the lower extremities, throwing-related shoulder and elbow injuries have also been reported to be significantly associated with dysfunction of the trunk and lower extremities, including hip ROM deficits, abnormal foot posture, and dynamic balance deficits [14,15,16,17,18,19,20].

A prospective study of youth baseball reported that shoulder external and internal rotation strength was associated with elbow injuries [13]. On the other hand, another study found no association between normalized elbow and shoulder muscle strength adjusted for height in school-aged children [21], indicating that no consensus has been reached. In terms of flexibility, a relationship between flexibility and injury has been reported in elementary school children [17,18,19,20]. Therefore, focusing on flexibility, the relationship between the ankle joint and throwing injuries has been unclear; however, in a recent prospective study in elementary school children, Shitara et al. [22] found that a lack of ankle dorsiflexion ROM in the back leg was a risk factor for shoulder and elbow injuries. Focusing on this aspect may enable field testing to predict the risk of throwing injuries. However, in the functional movement screen (FMS), one of the field tests, it has been reported that the FMS total score did not predict injury because it was not significantly associated with core stability, muscle strength, or muscle flexibility in elite college baseball players [23]. Moreover, the FMS total score cannot be used for injury prevention in high school baseball players because of its low sensitivity [24]. The FMS is a composite assessment consisting of seven movement tests, and the deep squat, which includes ankle movements, also requires trunk flexibility, which may prevent the extraction of risk factors for the ankle joints.

Yoshida et al. [25] evaluated ankle flexibility using the deep squatting test, which is also used in Japanese musculoskeletal examinations and has high reliability in measurement [26]. However, there have been very few studies that have prospectively examined the relationship between the deep squatting test and elbow injuries in elementary school baseball players. In addition, the deep squatting test is a simple method that does not require any measuring equipment, so athletes can perform it themselves. This test can also be graded according to the position of the upper extremities [25]. The forward test is performed with the upper extremity held forward, the middle test is performed with arms folded in front of the chest, and the backward test is performed with the hands clasped behind the hips. These difference results in a different center of gravity position, making the backward test more difficult than the forward and middle tests as a movement task because the ankle joint must be more dorsiflexed, and the center of gravity moves forward. To date, the deep squatting test has rarely been used to predict throwing elbow injuries; however, a detailed relationship with elbow injuries may be found by examining the test by difficulty levels. In fact, Okutani et al. [27] conducted a longitudinal study of the relationship between medial elbow injuries and the backward test in the deep squatting test in male elementary school students. However, because fifth and sixth grades were included among the subjects, and these subjects were limited to a subset of players who planned to continue playing baseball upon entering junior high school, it is possible that the characteristics of that age group could not have been extracted adequately. Therefore, the subjects of this study were considered to be baseball players who participated in pre-season medical checkups and were in elementary school during the season.

This study aimed to examine prospectively the relationship between the deep squatting test and medial elbow injuries in elementary school players who participated in youth baseball medical checkups and to determine the usefulness of the lower extremity field test in medial elbow injuries. In addition, to examine whether different difficulty levels of the squatting test affected the occurrence of medial elbow injuries, we conducted three different grades of deep squatting tests. Furthermore, since the flexibility of the lower extremities decreases during the growth period, we examined the relationship between the deep squatting test and medial elbow injuries by grade in elementary school.

## 2. Materials and Methods

### 2.1. Participants

**Figure 1:**
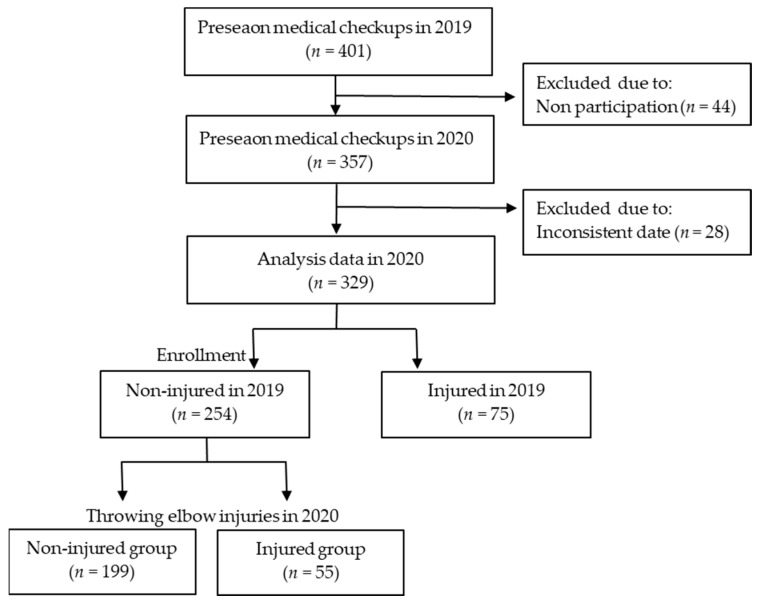
Flow chart of the players included in this study.

We provide annual preseason medical checkups for young baseball players who belong to a prefecture baseball federation for early detection of physical problems. In this longitudinal study, we recruited young male and female baseball players aged between 7 and 11 years during preseason medical checkups in 2019 and 2020 after explaining the study in detail. Players who were in Japanese elementary school during the season and who participated in preseason medical checkups in both 2019 and 2020 were included in this study (Figure 1). Those who had no problems during the 2019 medical checkups were considered. The exclusion criteria were as follows: (1) participants with lacked data for the deep squatting test; and (2) participants having a positive ultrasonography examination or physical examination in preseason medical checkups in 2019. The final sample size included in the analysis was 254 participants (Figure 1), which visually represents the study flow as a flowchart.

To calculate the required sample size for the χ^2^ test, we used a medium effect size of 0.3 (w), a significance level of 0.05 (α), a power of 0.8 (β), and a 2 × 3 contingency table, resulting in a required sample size of 108 participants. To further account for potential attrition, we estimated an additional 10% non-participation in the following year, 10% data loss, and 30% exclusion due to positive medial elbow injuries. This required recruiting more than 200 participants.

Before the experiment, the purpose and procedure were explained to the participants and their parents or guardians, and written informed consent was obtained in advance from both the participants and their parents or guardians. This study was also conducted with the approval of the Ethics Committee of the Kyoto Institute of Technology and in accordance with the Declaration of Helsinki (Protocol 15 Number 2018).

### 2.2. Medical Checkups

Preseason medical checkups in 2019 were conducted in December 2018, and preseason medical checkups in 2020 were conducted in December 2019 and February 2020. Preseason medical checkups in 2019 were performed as baseline medical examinations to evaluate the elbow and lower extremities. The following parameters were evaluated: height, weight, ultrasonography examination, physical examination, and the deep squatting test. The evaluation parameters in 2020 were ultrasonography examination and physical examination.

### 2.3. Ultrasonography Examination and Physical Examination

The examination included an ultrasonography of the medial elbow and a physical examination by an orthopedic surgeon, a clinical laboratory technician, and a physical therapist. Ultrasonography examination was performed according to the method of Harada et al. [28] The presence or absence of irregularity of the medial epicondyle of the humerus was checked, and those who showed irregularity, separation, or protrusion were classified as positive for ultrasonography examination. A physical examination, medial elbow tenderness test, and valgus stress test were also performed. In the medial elbow tenderness test, the medial epicondyle of the humerus was manually compressed, and the pain was defined as positive. In the valgus stress test, the elbow joint was immobilized, the forearm was placed in the external rotation position, and the elbow joint was flexed at three different angles (30°, 60°, and 90°) while external rotation was dynamically forced. The valgus stress test result was considered positive when the pain was observed at any angle. Any study participant who exhibited tenderness of the medial elbow or valgus stress was also classified as positive for physical examination. Ultrasonography examination is widely used for the evaluation of elbow injuries and to assess morphological abnormalities of the bones and soft tissues in the elbow. Ultrasonography examination is capable of identifying previous elbow injuries caused by pitching irrespective of whether the elbow is painful when the scan is performed. In contrast, the medial elbow tenderness test and the valgus stress test are physical assessments used to confirm pain during evaluation [29]. In this study, we combined the assessment of chronic elbow stress and acute pain to examine new injuries that occurred within one year. Therefore, medial elbow injuries were defined as a positive ultrasonography or physical examination during the preseason medical checkups in 2020. To identify and analyze variations between injured and non-injured players, participants were classified into injured and non-injured groups based on the 2020 preseason medical checkups.

### 2.4. Deep Squatting Test

For the field test, three types of squatting tests were conducted based on the methods described by Yoshida et al. [25]. The starting extremities position for the test was the standing position with the medial sides of both feet and knees grounded. The participants were asked to sit in a deep squatting posture with their heels down and to maintain the posture for more than 5 s with the upper extremities held forward in the forward deep squatting test, with arms folded in front of the chest in the middle deep squatting test and with hands folded at the waist in the backward deep squatting test. The criteria were as follows: (1) inability to hold the posture until the hips and heels were close together, (2) both knees apart, (3) heels off the ground, and (4) failure to hold for 5 s. Tests were taken barefoot.

### 2.5. Questionnaire

A questionnaire survey was used to investigate the participants’ competitive experience, the frequency of training sessions per week, and the maximum full-strength pitch count on a single day during the week. The frequency of training sessions per week was scored on a five-point scale, ranging from one to seven sessions, and the maximum full-strength pitch count in a single day during the week was scored on an eight-point scale, ranging from less than 40 pitches to 100 pitches or more.

### 2.6. Statistical Analysis

Baseline characteristics are reported as mean ± standard deviation. Group differences in the baseline characteristics between injured and non-injured groups were evaluated using an independent two-tailed *t*-test. We used the chi-square test to investigate the difference between the grades in the percentage of medial elbow injuries and the percentage of successful squatting tests. Residual analysis was performed to identify specific cells that contributed the most to the chi-square test results. The chi-square test was used to examine the impact of the results of the deep squatting test on the results of the medical checkup, which were examined longitudinally. All statistical analyses were performed using SPSS version 27 (IBM Japan, Ltd., Tokyo, Japan). All statistical tests were two-sided with a significance level set at *p* = 0.05.

## 3. Results

### 3.1. Characteristics of Participants

A total of 401 players belonging to grades 2–4 participated in the 2019 preseason medical checkups, of whom 357 also participated in the 2020 preseason medical checkups. Of these, 254 players (241 males, 13 females) who were non-injured in 2019 were enrolled in the study, excluding 28 with missing data.

No significant differences were observed in age, height, or weight between the injured and non-injured groups (Table 1). The competitive experience of the participants was 1.5 ± 1.0 years. The weekly frequency of training was twice per week for 75.8% of participants (Table 2).

### 3.2. Medial Elbow Injuries

The incidence of medial elbow injuries was 21.7% (*n* = 55; Figure 1). There were no significant differences in medial elbow injuries between the grades (Table 3). The incidence of ultrasound findings was 17.3%, with significant differences observed between grades (χ^2^ = 6.669, *p* = 0.036). The incidence of the medial elbow tenderness test was 3.1%, with no significant differences observed between grades. The incidence of the Valgus stress test was 4.3%, with no significant differences observed between grades (Table 3). In the ultrasonography examination, medial elbow tenderness test, and Valgus stress test, there were participants who tested positive in multiple assessments.

### 3.3. Deep Squatting Test

The results of the deep squatting tests conducted during the first year are listed in Table 4. The success rate of the squatting test was 86.6% (*n* = 220) for the forward test, 81.1% (*n* = 206) for the middle test, and 55.5% (*n* = 141) for the backward test. There were no significant differences between grades in the forward, middle, and backward tests.

The success rate in the forward, middle, and backward squatting tests by the medical checkup group is shown by all and grade (Table 5). For the forward test, there was no significant difference in success rates between the non-injured and injured groups, but for the middle and backward tests, the injured group had significantly lower success rates than the non-injured group. (forward test: χ^2^ = 2.649, *p* = 0.104; middle test: χ^2^ = 6.608, *p* = 0.010; backward test: χ^2^ = 10.422, *p* = 0.001). By grade, there was no significant difference in either test between the non-injured and injured groups in the fourth grade (forward test: χ^2^ = 1.073, *p* = 0.300; middle test: χ^2^ = 0.123, *p* = 0.726; backward test: χ^2^ = 0.629, *p* = 0.410). However, in the second and third grades, there were significant differences between groups on all tests (forward test, second grade: χ^2^ = 8.423, *p* = 0.004; forward test, third grade: χ^2^ = 4.180, *p* = 0.041; middle test, second grade: χ^2^ = 12.452, *p* < 0.001; middle test, third grade: χ^2^ = 5.431, *p* = 0.020; backward test, second grade: χ^2^ = 13.844, *p* < 0.001; and backward test, third grade: χ^2^ = 12.312, *p* < 0.001).

## 4. Discussion

We prospectively investigated the relationship between the deep squatting test and medial elbow injuries in elementary school baseball players. In this study, the success rate of the deep squatting test was significantly lower in the injured group than in the non-injured group. Shitara et al. [22] found that a lack of ankle dorsiflexion ROM is a risk factor for shoulder and elbow injuries. In the present study, we performed a deep squatting test as a field test to evaluate ankle flexibility and found that it was associated with medial elbow injuries. To our knowledge, this is the first prospective study to provide evidence that the deep squatting test by the difficulty level can predict the risk of subsequent medial elbow injuries in elementary school players. This evidence may contribute to the development of new strategies for throwing injury prevention programs.

While this study cannot definitively determine why the inability to squat increases the risk of injury, there are some potential explanations. The throwing motion is known to be a whole-body movement based on a kinetic chain involving the lower extremities, trunk, and upper extremities [30]. The force generated by the lower extremities of the body during throwing is transferred via the kinetic chain through the core, shoulder, elbow, and ultimately, the hand before ball release [31,32,33]. Previous studies have suggested that disruptions in the kinetic chain may lead to upper extremity injuries, with reports indicating that a reduction in the range of motion of lower extremities joints may specifically impact throwing mechanics [18,19,20,34]. Hamano et al. [20] conducted a prospective study on elementary and junior high school students, identifying a reduced hip range of motion as a risk factor for shoulder and elbow injuries. An inadequate hip range of motion may alter throwing mechanics, leading to a reduced transfer of energy from the lower to the upper extremities, thereby increasing the load on the upper extremities [19]. Specifically, limited hip ROM has been suggested to increase valgus torque on the elbow, potentially elevating the risk of medial elbow injuries.

Shitara et al. [22] found that a lack of ankle dorsiflexion ROM in the back leg during pitching motion is a risk factor for shoulder and elbow injuries. Additionally, they indicated that dysfunction in the ankle joint significantly impacts kinetic changes in adjacent segments or beyond. When transferring accumulated force into kinetic energy through the loading of weight in the foot and ankle during the transition from the wind-up phase to the arm-cocking phase, appropriate dorsiflexion of the ankle on the back leg is required. Consequently, even a slight restriction in dorsiflexion of the rear leg ankle may have resulted in a loss of kinetic energy, suggesting a possible contribution to the development of throwing-related injuries. The results of these longitudinal studies suggest that a limited range of motion in the hip and ankle may increase valgus torque on the elbow from a kinetic chain perspective, thereby potentially elevating the risk of medial elbow injuries. In this study, a squatting test was conducted to provide a simple evaluation of ankle flexibility. If an individual is unable to perform the squatting test, it is possible that reduced ankle flexibility disrupts the kinetic chain, leading to compensatory movements that increase stress on the medial elbow. In other words, compensatory motions resulting from an inability to squat can contribute to a breakdown in throwing form, potentially increasing the risk of injuries. This suggests that the inability to perform a squat may elevate the risk of medial elbow injuries. According to previous studies, the total FMS score has been reported to be ineffective in predicting injury in high school and college baseball players [23,24]. Because the FMS is a composite score composed of seven basic movement tests, it may not be sufficient to identify specific movement patterns associated with poor throwing form. Additionally, the deep squat component of the FMS, which requires holding a bar overhead with extended arms, demands trunk strength and flexibility, potentially limiting its ability to identify risk factors for the ankle joints.

In addition, we attempted to examine the relationship between the difficulty of squatting and elbow injuries. In this study, we performed three types of deep squatting tests. The results showed that the backward test was better at predicting medial elbow injuries than the forward and middle tests. The success rates of the forward and middle tests were higher than that of the backward test. Yoshida et al. [25] reported that the ankle dorsiflexion ROM in a cohort of young males and females during forward, middle, and backward squatting was 41 ± 4.6°, 46 ± 4.9°, and 49 ± 8.6°, respectively, with a significant difference observed between forward and backward squatting. Hence, performing the backward squatting requires more flexibility of ankle dorsiflexion. Additionally, Shitara et al. [22] found that a lack of ankle dorsiflexion ROM in the back leg is a risk factor for shoulder and elbow injuries. Although our results showed significantly more backward and middle tests than forward tests, considering the previous results [22,25], the forward and middle tests cannot predict medial elbow injuries. The backward test requires higher flexibility and a higher level of ankle flexibility has been associated with elbow injuries. Thus, the backward test is a more useful field test for predicting medial elbow injuries in elementary school players.

We also examined the results by grade level and found grade differences in the relationship between the deep squatting test and medial elbow injuries. Previous studies have reported considerable variability in throwing mechanics among young athletes [35]. The participants in this study were elementary school students from second to fourth grade, training approximately twice per week. Considering the participants’ age range and training frequency, it seems unlikely that their throwing mechanics are fully developed or stable. In this study, despite the presumed instability in throwing mechanics among second and third graders, a relationship between deep squatting test and elbow injuries was found. This suggests that limited ankle flexibility may disrupt the kinetic chain and lead to improper throwing mechanics, potentially resulting in elbow injuries. In contrast, in the fourth grade, there was no significant difference in the success rate between groups in the forward, middle, and backward tests. In the backward test, 58% of players in the injured group were able to squat, suggesting that, beyond disruptions in the kinetic chain due to reduced ankle flexibility, other factors may also have contributed to poor throwing mechanics. According to previous studies, excessive lateral trunk flexion, early trunk rotation, a low elbow position during throwing, or a reliance on arm motion alone have been reported as causes of poor form [36,37,38]. These factors may also be at play.

Previous studies have reported a significant association between trunk or lower extremity dysfunction and elbow or shoulder injuries in overhead athletes, such as volleyball, handball, and tennis players [34,39]. Specifically, young overhead athletes with pain in the hip, knee, or foot are more likely to experience elbow or shoulder pain than those without pain [34], suggesting a critical link between dysfunction of trunk or lower extremities and upper extremity injuries. Therefore, the association between the squatting test and medial elbow injuries observed in this study may be applicable to other overhead sports, including tennis, handball, and volleyball, in addition to baseball.

This study had several limitations. Because the ROM of the lower extremity was not measured, it was not possible to determine which parts of the lower extremity were associated with the medial elbow. In this study, we did not observe pitching form, so we could not determine whether disruptions in the kinetic chain due to reduced ankle flexibility led to poor form and injury in the fourth grade, or whether pitching form problems caused by other factors were responsible for the injuries. Future research should clarify how the deep squatting test influences pitching form and injury occurrence. In particular, examining differences in pitching form and injury risk based on the ability to perform the deep squatting test may demonstrate the utility of the test, even at grade levels where no associations were observed in this study. Furthermore, this work highlights the importance of training methods, skill development, and technical education tailored to elementary school children. Addressing these limitations in future studies will strengthen understanding in this area.

## 5. Conclusions

This study aimed to examine prospectively the relationship between the deep squatting test and medial elbow injuries in elementary school players who participated in youth baseball medical checkups and to determine the usefulness of the lower extremity field test in medial elbow injuries. The incidence of new medial elbow injuries among elementary school baseball players was 21.7%. Among elementary school players, the success rate of squatting tests was significantly lower in the injured group than in the non-injured group. The difficulty results showed that the backward test was more likely to predict obstacles than the forward and middle tests. Furthermore, differences were found between grades, with a relationship between the squatting test and medial elbow injuries in the second and third grades but not in the fourth grade. The squatting test may be useful as a field test for lower extremity flexibility that can be performed by athletes and instructors, as it can predict the risk of subsequent medial elbow injuries to some extent.

## Figures and Tables

**Table 1:** Baseline characteristics of study participants.

	Medical Check-Ups				
	Non-Injured		Injured		
	Mean	SD	Mean	SD	*p*
Age (year)	8.9	0.9	9.0	0.8	0.644
Height (cm)	132.6	6.8	133.1	7.2	0.753
Weight (kg)	29.2	5.1	29.5	4.9	0.154

SD: standard deviation.

**Table 2:** Frequency of training and pitch count by grades in elementary school.

	All	Second-Grade	Third-Grade	Fourth-Grade
Competitive experience	1.5 ± 1.0	0.9 ± 0.7	1.5 ± 0.9	1.8 ± 1.1
Weekly frequency of training				
1 day	1 (0.4)	0 (0.0)	1 (1.1)	0 (0.0)
2 days	188 (75.8)	46 (83.6)	69 (72.6)	73 (74.5)
3–4 days	41 (16.5)	8 (14.5)	17 (17.9)	16 (16.3)
5–6 days	9 (3.6)	0 (0.0)	6 (6.3)	3 (3.1)
7 days	9 (3.6)	1 (1.8)	2 (2.1)	6 (6.1)
Full-strength pitch count in one day				
<40	85 (36.2)	24 (45.3)	33 (36.3)	28 (30.8)
<50	48 (20.4)	13 (24.5)	19 (20.9)	16 (17.6)
<60	25 (10.6)	4 (7.5)	10 (11.0)	11 (12.1)
<70	22 (9.4)	3 (5.7)	8 (8.8)	11 (12.1)
<80	10 (4.3)	0 (0.0)	6 (6.6)	4 (4.4)
<90	2 (0.9)	1 (1.9)	1 (1.1)	0 (0.0)
<100	21 (8.9)	4 (7.5)	7 (7.2)	10 (11.0)
≥100	22 (9.4)	4 (7.5)	7 (7.2)	11 (12.1)

Values for competitive experience are the mean ± standard deviation. The values for the weekly frequency of training and full-strength pitch count in one day are presented as frequencies. Figures in parentheses show the percentage of the participant or grade universe.

**Table 3:** Medial elbow injuries by grades in elementary school.

	All	Second-Grade	Third-Grade	Fourth-Grade	χ^2^	*p*
*n*	254	55	97	102		
Medial elbow injuries	55 (21.7)	6 (10.9)	23 (23.7)	26 (25.5)	4.870	0.088
Ultrasound findings	44 (17.3)	4 (7.3)	16 (16.5)	24 (23.5)	6.669	0.036 *
Medial elbow tenderness test	8 (3.1)	1 (1.8)	5 (5.2)	2 (2.0)	2.071	0.355
Valgus stress test	11 (4.3)	2 (3.6)	7 (7.2)	2 (2.0)	3.396	0.183

Figures in parentheses show the percentage of the participant or grade universe. *, *p* < 0.05.

**Table 4:** Successful participants in forward, middle, and backward squatting tests by grades.

	All	Second-Grade	Third-Grade	Fourth-Grade	χ^2^	*p*
*n*	254	55	97	102		
Forward test	220 (86.6)	48 (87.3)	84 (86.6)	88 (86.3)	0.031	0.985
Middle test	206 (81.1)	46 (83.6)	76 (78.4)	84 (82.4)	0.814	0.666
Backward test	141 (55.5)	37 (67.3)	52 (53.6)	52 (51.0)	4.071	0.131

Figures in parentheses show the percentage of the participant or grade universe.

**Table 5:** Number of successful participants in forward, middle, and backward squatting tests by medical check-up groups.

	Medical Check-Ups				
	Non-Injured	Injured	χ^2^	*p*	
All					
*n*	199	55			
Forward test	176 (88.4)	44 (80.0)	2.649	0.104	
Middle test	168 (84.4)	38 (69.1)	6.608	0.010	*
Backward test	121 (60.8)	20 (36.4)	10.422	0.001	**
Second-grade					
*n*	49	6			
Forward test	45 (91.8)	3 (50.0)	8.423	0.004	**
Middle test	44 (89.8)	2 (33.3)	12.452	<0.001	**
Backward test	37 (75.5)	0 (0.0)	13.844	<0.001	**
Third-grade					
*n*	74	23			
Forward test	67 (90.5)	17 (73.9)	4.180	0.041	*
Middle test	62 (83.8)	14 (60.9)	5.431	0.020	*
Backward test	47 (63.5)	5 (21.7)	12.312	<0.001	**
*n*	76	26			
Forward test	64 (84.2)	24 (92.3)	1.073	0.300	
Middle test	62 (81.6)	22 (84.6)	0.123	0.726	
Backward test	37 (48.7)	15 (57.7)	0.629	0.428	

Figures in parentheses show the percentage of the participant universe. *, *p*< 0.05; **, *p* < 0.01

## Data Availability

All experimental data files are available from the figshare database. https://doi.org/10.6084/m9.figshare.26885596.v1 (accessed on 2 September 2024).

## References

[1] Kivlan B.R., Carcia C.R., Clemente F.R., Phelps A.L., Martin R.L. (2013). Reliability and validity of functional performance tests in dancers with hip dysfunction. Int. J. Sports Phys. Ther..

[2] Cook G., Burton L., Hoogenboom B.J., Voight M. (2014). Functional movement screening: The use of fundamental movements as an assessment of function—Part 1. Int. J. Sports Phys. Ther..

[3] Yoshida M., Aoki N., Taniguchi K., Yoshida M., Katayose M. (2018). Kinematic analysis of the ankle joint on the side-hop test in subjects with ankle sprains. Transl. Sports Med..

[4] Uchida T., Matsumoto S., Komatsu M., Noda Y., Ishida M., Tsukuda M., Nakayama R., Takeda Y., Hirakawa R., Muto K. (2016). Relationships between throwing injuries and functional movement screen in junior high school baseball players. Jpn. J. Phys. Fit. Sports Med..

[5] Plisky P.J., Rauh M.J., Kaminski T.W., Underwood F.B. (2006). Star Excursion Balance Test as a predictor of lower extremity injury in high school basketball players. J. Orthop. Sports Phys. Ther..

[6] Dallinga J.M., Benjaminse A., Lemmink K.A. (2012). Which screening tools can predict injury to the lower extremities in team sports? A systematic review. Sports Med..

[7] Hegedus E.J., McDonough S., Bleakley C., Cook C.E., Baxter G.D. (2015). Clinician-friendly lower extremity physical performance measures in athletes: A systematic review of measurement properties and correlation with injury, part 1. The tests for knee function including the hop tests. Br. J. Sports Med..

[8] Shanley E., Rauh M.J., Michener L.A., Ellenbecker T.S., Garrison J.C., Thigpen C.A. (2011). Shoulder range of motion measures as risk factors for shoulder and elbow injuries in high school softball and baseball players. Am. J. Sports Med..

[9] Shanley E., Kissenberth M.J., Thigpen C.A., Bailey L.B., Hawkins R.J., Michener L.A., Tokish J.M., Rauh M.J. (2015). Preseason shoulder range of motion screening as a predictor of injury among youth and adolescent baseball pitchers. J. Shoulder Elbow Surg..

[10] Wilk K.E., Macrina L.C., Fleisig G.S., Porterfield R., Simpson C.D., Harker P., Paparesta N., Andrews J.R. (2011). Correlation of glenohumeral internal rotation deficit and total rotational motion to shoulder injuries in professional baseball pitchers. Am. J. Sports Med..

[11] Shitara H., Kobayashi T., Yamamoto A., Shimoyama D., Ichinose T., Tajika T., Osawa T., Iizuka H., Takagishi K. (2017). Prospective multifactorial analysis of preseason risk factors for shoulder and elbow injuries in high school baseball pitchers. Knee Surg. Sports Traumatol. Arthrosc..

[12] Shitara H., Tajika T., Kuboi T., Ichinose T., Sasaki T., Hamano N., Endo F., Kamiyama M., Miyamoto R., Kakase K. (2021). Asymptomatic Medial Elbow Ultrasound Abnormality in Youth Baseball Players Is an Independent Risk Factor for Elbow Injury: A Prospective Cohort Study. Orthop. J. Sports Med..

[13] Harada M., Takahara M., Mura N., Sasaki J., Ito T., Ogino T. (2010). Risk factors for elbow injuries among young baseball players. J. Shoulder Elbow Surg..

[14] Feigenbaum L.A., Roach K.E., Kaplan L.D., Lesniak B., Cunningham S. (2013). The association of foot arch posture and prior history of shoulder or elbow surgery in elite-level baseball pitchers. J. Orthop. Sports Phys. Ther..

[15] Hannon J., Garrison J.C., Conway J. (2014). Lower extremity balance is improved at time of return to throwing in baseball players after an ulnar collateral ligament reconstruction when compared to pre-operative measurements. Int. J. Sports Phys. Ther..

[16] Garrison J.C., Arnold A., Macko M.J., Conway J.E. (2013). Baseball players diagnosed with ulnar collateral ligament tears demonstrate decreased balance compared to healthy controls. J. Orthop. Sports Phys. Ther..

[17] Sekiguchi T., Hagiwara Y., Yabe Y., Tsuchiya M., Itaya N., Yoshida S., Yano T., Sogi Y., Suzuki K., Itoi E. (2020). Restriction in the hip internal rotation of the stride leg is associated with elbow and shoulder pain in elite young baseball players. J. Shoulder Elbow Surg..

[18] Endo Y., Sakamoto M. (2014). Correlation of shoulder and elbow injuries with muscle tightness, core stability, and balance by longitudinal measurements in junior high school baseball players. J. Phys. Ther. Sci..

[19] Saito M., Kenmoku T., Kameyama K., Murata R., Yusa T., Ochiai N., Kijima T., Takahira N., Fukushima K., Ishige N. (2014). Relationship Between Tightness of the Hip Joint and Elbow Pain in Adolescent Baseball Players. Orthop. J. Sports Med..

[20] Hamano N., Shitara H., Tajika T., Ichinose T., Sasaki T., Kuboi T., Shimoyama D., Kamiyama M., Miyamoto R., Endo F. (2021). Relationship between the Lower Limb Function and Shoulder and Elbow Injuries in Elementary School Baseball Pitchers. Prog. Rehabil. Med..

[21] Sakata J., Nakamura E., Suzukawa M., Akaike A., Shimizu K., Aoki H. (2016). The Prospective Study of Risk Factors for the Medial Baseball Elbow in the Little League Players. Jpn. J. Orthop. Sports Med..

[22] Shitara H., Tajika T., Kuboi T., Ichinose T., Sasaki T., Hamano N., Endo F., Kamiyama M., Miyamoto R., Nakase K. (2021). Risk factor for elbow symptom manifestation in young baseball players with asymptomatic medial elbow abnormalities: A prospective cohort study. Sci. Rep..

[23] Liang Y.P., Kuo Y.L., Hsu H.C., Hsia Y.Y., Hsu Y.W., Tsai Y.J. (2019). Collegiate baseball players with more optimal functional movement patterns demonstrate better athletic performance in speed and agility. J. Sports Sci..

[24] Lee C.L., Hsu M.C., Chang W.D., Wang S.C., Chen C.Y., Chou P.H., Chang N.J. (2018). Functional movement screen comparison between the preparative period and competitive period in high school baseball players. J. Exerc. Sci. Fit..

[25] Yoshida M., Yoshida M. (2014). The Relationship between the Deep Squatting Test and range of motion of the ankle joint. Bull. Hokusho Univ. Sch. Lifelong Sport.

[26] Uchio Y., Takahashi T., Muto Y. (2018). Musculoskeletal Examination of School.

[27] Okutani T., Maruyama S., Matsui T., Tanaka M., Kida N., Takeshima M., Morihara T. (2024). Longitudinal survey on the relationship between simple physical function test and medial elbow injuries in medical checkup for young baseball players: Evaluation using a simple lower limb functional test. Jpn. J. Clin. Sports Med..

[28] Harada M., Takahara M., Sasaki J., Mura N., Ito T., Ogino T. (2006). Using sonography for the early detection of elbow injuries among young baseball players. AJR Am. J. Roentgenol..

[29] Gonno M., Kida N., Nomura T., Matsui T., Azuma Y., Hiramoto M., Hashimoto R., Miyazaki T., Tanaka M., Watanabe Y. (2022). Relationship between Standing Trunk Extension Angle and Medial Elbow Injuries in Young Baseball Pitchers. Int. J. Environ. Res. Public Health.

[30] Sauers E.L., Huxel Bliven K.C., Johnson M.P., Falsone S., Walters S. (2014). Hip and glenohumeral rotational range of motion in healthy professional baseball pitchers and position players. Am. J. Sports Med..

[31] Fleisig G.S., Escamilla R.F., Barrentine S.W., Andrews J.R., Zarins B., Wilk K.E. (1998). Biomechanics of pitching: Mechanism and motion analysis. Injuries in Baseball.

[32] Thompson S.F., Guess T.M., Plackis A.C., Sherman S.L., Gray A.D. (2018). Youth Baseball Pitching Mechanics: A Systematic Review. Sports Health.

[33] Wilk K.E., Meister K., Fleisig G., Andrews J.R. (2000). Biomechanics of the overhead throwing motion. Sports Med. Arthrosc. Rev..

[34] Sekiguchi T., Hagiwara Y., Momma H., Tsuchiya M., Kuroki K., Kanazawa K., Yabe Y., Yoshida S., Koide M., Itaya N. (2017). Coexistence of Trunk or Lower Extremity Pain with Elbow and/or Shoulder Pain among Young Overhead Athletes: A Cross-Sectional Study. Tohoku J. Exp. Med..

[35] Fleisig G., Chu J., Weber A., Andrews J. (2009). Variability in baseball pitching biomechanics among various levels of competition. Sports Biomech..

[36] Davis J.T., Limpisvasti O., Fluhme D., Mohr K.J., Yocum L.A., Elattrache N.S., Jobe F.W. (2009). The effect of pitching biomechanics on the upper extremity in youth and adolescent baseball pitchers. Am. J. Sports Med..

[37] Oyama S., Waldhelm A.G., Sosa A.R., Patel R.R., Kalinowski D.L. (2017). Trunk Muscle Function Deficit in Youth Baseball Pitchers With Excessive Contralateral Trunk Tilt During Pitching. Clin. J. Sport. Med..

[38] Solomito M.J., Garibay E.J., Golan E., Nissen C.W. (2021). Elbow flexion post ball release is associated with the elbow varus deceleration moments in baseball pitching. Sports Biomech..

[39] Radwan A., Francis J., Green A., Kahl E., Maciurzynski D., Quartulli A., Schultheiss J., Strang R., Weiss B. (2014). Is there a relation between shoulder dysfunction and core instability?. Int. J. Sports Phys. Ther..

